# Sequences of complete human cytomegalovirus genomes from infected cell cultures and clinical specimens

**DOI:** 10.1099/vir.0.015891-0

**Published:** 2010-03

**Authors:** Charles Cunningham, Derek Gatherer, Birgitta Hilfrich, Katarina Baluchova, Derrick J. Dargan, Marian Thomson, Paul D. Griffiths, Gavin W. G. Wilkinson, Thomas F. Schulz, Andrew J. Davison

**Affiliations:** 1 MRC Virology Unit, Institute of Virology, University of Glasgow, Church Street, Glasgow G11 5JR, UK; 2 Institute of Virology, Hannover Medical School, Carl-Neuberg-Strasse 1, D-30625 Hannover, Germany; 3 The GenePool, Ashworth Laboratories, King's Buildings, University of Edinburgh, Edinburgh EH9 3JT, UK; 4 Centre for Virology, University College Medical School, Royal Free Campus, Rowland Hill Street, Hampstead, London NW3 2QG, UK; 5 Department of Medical Microbiology, Tenovus Building, School of Medicine, Cardiff University, Heath Park, Cardiff CF14 4XX, UK

## Abstract

We have assessed two approaches to sequencing complete human cytomegalovirus (HCMV) genomes (236 kbp) in DNA extracted from infected cell cultures (strains 3157, HAN13, HAN20 and HAN38) or clinical specimens (strains JP and 3301). The first approach involved amplifying genomes from the DNA samples as overlapping PCR products, sequencing these by the Sanger method, acquiring reads from a capillary instrument and assembling these using the Staden programs. The second approach involved generating sequence data from the DNA samples by using an Illumina Genome Analyzer (IGA), processing the filtered reads by reference-independent (*de novo*) assembly, utilizing the resulting sequence to direct reference-dependent assembly of the same data and finishing by limited PCR sequencing. Both approaches were successful. In particular, the investigation demonstrated the utility of IGA data for efficiently sequencing genomes from clinical samples containing as little as 3 % HCMV DNA. Analysis of the genome sequences obtained showed that each of the strains grown in cell culture was a mutant. Certain of the mutations were shared among strains from independent clinical sources, thus suggesting that they may have arisen in a common ancestor during natural infection. Moreover, one of the strains (JP) sequenced directly from a clinical specimen was mutated in two genes, one of which encodes a proposed immune-evasion function, viral interleukin-10. These observations imply that HCMV mutants exist in human infections.

## INTRODUCTION

Human cytomegalovirus (HCMV; species *Human herpesvirus 5*) has the largest genome of any known human virus, at 236 kbp in size (Dolan *et al.*, 2004). The genome is a linear, double-stranded DNA molecule consisting of two unique regions, each flanked by inverted repeats. The structure is represented as *ab*-U_L_-*b*′*a*′*c*′-U_S_-*ca*, where U_L_ and U_S_ denote the long and short unique regions and *ba*/*b*′*a*′ and *ca*/*c*′*a*′ indicate the inverted repeats. In wild-type HCMV strain Merlin (GenBank accession no. AY446894), the sizes of U_L_ and U_S_ are 193 019 and 35 482 bp, respectively, and those of *a*/*a*′, *b*/*b*′ and *c*/*c*′ are 578, 746 and 1959 bp, respectively.

Many studies have shown that HCMV strains are impressively divergent in a subset of genes encoding membrane-associated or secreted proteins (e.g. Dolan *et al.*, 2004; Murphy *et al.*, 2003; Pignatelli *et al.*, 2004). Each of these hypervariable genes exists as several highly diverged clusters of alleles, with a much lower level of allelic variation evident within individual clusters. The sequences of particular alleles are stable on short timescales in patients (Hassan-Walker *et al.*, 2004; Stanton *et al.*, 2005) and during cell culture (Lurain *et al.*, 2006). These observations suggest that hypervariation is a result of immune selection and that the allelic clusters have a long history, perhaps having emerged during the evolution of populations of early humans or their predecessors (Bradley *et al.* 2008; Dolan *et al.*, 2004). Also, there is extensive evidence that recombination has occurred during HCMV evolution, and that HCMV infections frequently involve multiple strains (e.g. Arav-Boger *et al.*, 2005; Bates *et al.*, 2008; Bradley *et al.*, 2008; Mattick *et al.*, 2004; Puchhammer-Stöckl & Görzer, 2006; Rasmussen *et al.*, 2003; Yan *et al.*, 2008). These factors add significant complexities to assessments of the associations between the genetic constitution of HCMV strains and disease outcome. Further work investigating HCMV genomes in clinical material is required in order to facilitate evaluations of the extent of strain variation, the occurrence of mixed infections and the biological correlates of particular genetic configurations.

All of the HCMV genome sequences published to date (Table 1) have been derived from strains grown in cell culture, which is known to exert selective pressure and induce genetic adaptations, some of which are extensive (Cha *et al.*, 1996; Dolan *et al.*, 2004; Prichard *et al.*, 2001). With two recent exceptions discussed below, these sequences were determined by random shotgun cloning of standard bacterial plasmid libraries, bacterial artificial chromosomes (BACs) or purified virion DNA, followed by Sanger sequencing. To sequence HCMV genomes in clinical material, it is important to avoid cell culture, because of its mutagenic effects and the likelihood that not all strains present will transfer successfully from a specimen. Also, it is important not to utilize bacterial plasmids or BACs, since such clones may not fully represent the virus populations from which they originated. In this paper, we explore two approaches to sequencing complete HCMV genomes from infected cell cultures and clinical specimens that meet these requirements, and that also cope with the presence of high proportions of cellular DNA in such substrates.

The first approach (PCR sequencing) involved generating numerous, overlapping PCR products from HCMV genomes and analysing them by Sanger sequencing. This overcomes the presence of cellular DNA by amplifying HCMV sequences selectively. It amounts to an extension of a method that has been used by many researchers to analyse selected regions of HCMV genomes, in some studies to a substantial extent (Brondke *et al.*, 2007; Dolan *et al.*, 2004).

The use of an Illumina Genome Analyzer (IGA) formed the basis of the second approach (IGA sequencing). This high-throughput instrument generates such a large number of sequence reads that a virus genome can be sequenced even when diluted substantially by cellular DNA. Genome sequences may be assembled from IGA data by two strategies, each of which has advantages. Reference-dependent assembly (DA) involves aligning the reads with a reference sequence, whereas reference-independent (*de novo*) assembly (IA) deduces long contiguous sequences (contigs) from the reads alone. DA is likely to cover a genome more comprehensively than IA, and in a manner that does not contain erroneously joined regions. On the other hand, IA, by not requiring a reference, is not biased by presumptions made about the target sequence.

We used DA previously to sequence strain AD169 variant UC (varUC) and strain Towne varL from samples that contained high proportions of HCMV DNA (Bradley *et al.*, 2009). These are the two exceptions mentioned above (Table 1). In the present paper, we have assessed the utility of DA and IA for sequencing HCMV genomes from infected cell cultures and clinical specimens containing much lower proportions of HCMV DNA. We developed a strategy for analysing HCMV genomes by IGA sequencing that consists of processing filtered reads by IA, then using the resulting sequence as a reference in DA in order to inspect the read alignment and correct any errors, and finally finishing by limited PCR sequencing.

## RESULTS

### HCMV genomes sequenced

The sequences of six HCMV strains were determined, some by more than one method (Table 2). Strains 3157, JP and HAN13 were analysed by PCR sequencing, and strains HAN13, HAN20, HAN38 and 3301 by IGA sequencing. IGA data were assembled by two strategies, DA and IA, and, during the latter process, HCMV Gap4 contigs were aligned by using a cognate scaffold (IAC) or a generic scaffold (IAG). The analysis was supplemented by IAC of data reported previously for strains AD169 varUC and Towne varL (Bradley *et al.*, 2009).

### PCR sequencing

The PCR sequencing approach was successful and, as it has been used previously in many studies for analysing regions of HCMV genomes, it is not elaborated further. However, IGA sequencing is a novel approach that has been utilized with HCMV only for DA of strains AD169 varUC and Towne varL, and is expanded upon below.

### IGA sequencing: reference-dependent assembly

IGA sequences were assembled in the present study by DA for strains HAN13 and 3301 (Table 2). A summary of the results is provided in Table 3.

The reference used for strain HAN13 was generated from the genome determined by PCR sequencing, and, as anticipated, needed no improvements (i.e. the sequences derived by the two methods were identical). The reference used for strain 3301 was constructed by inserting strain 3301 sequences generated by PCR sequencing into a strain Merlin backbone. The strain 3301 PCR sequences had either been published previously (Dolan *et al.*, 2004; GenBank accession numbers are given below) or were produced for the present study. They totalled 57 977 bp (approx. 25 %) of the reference: 18 452 bp containing genes RL5A to UL11 and parts of flanking genes RL1 and UL13 (AY446862), 2820 bp containing genes UL73, UL74, UL74A and parts of flanking genes UL72 and UL75, 6096 bp containing gene UL111A and parts of flanking genes UL105 and UL112 (AY446874), 25 616 bp containing genes UL120 to UL150 and part of the flanking gene UL119 (AY446864), 2011 bp extending from AY446864 into the *b*′, *a*′ and part of the *c*′ inverted repeats, and 2982 bp containing genes US34 and US34A and part of gene US32. The read consensus was extracted from the DA and used as an improved reference, thus correcting mismatches due to components in the initial reference that had originated from strain Merlin. Residual gaps in the read consensus due to insertions/deletions (indels) or clustered mismatches were remedied by utilizing an alignment of previously determined HCMV sequences as a guide to producing a further improved reference by reiterative local assembly. DA using this reference left four small gaps in the read consensus. One was located at the left end of U_L_ (including the junction with the adjacent *b* inverted repeat), one in a (G+C)-rich region near the origin of DNA replication, and two in tandem, direct repeats of undetermined lengths (one a 3 nt reiteration and the other a 1 nt reiteration or homopolymeric tract). These gaps were resolved by PCR sequencing. At the conclusion of the assembly process, a total of 3 % of the reads aligned with the final sequence.

### IGA sequencing: reference-independent assembly

An example of the output of the earlier stages of IA is given for strain Towne varL in Table 4, and the output for this and the other strains at the later stages is listed in Table 3. Thus, for strain Towne, 11 filtered datasets, in addition to the unfiltered dataset assembled with two values of *k*, were employed in 13 assemblies (Table 4). A total of 2087 pooled IA contigs was generated (the sum of the ‘all contigs’ column in Table 4). From the 1664 pooled, non-redundant IA contigs derived from these, 96 Phrap contigs and then nine HCMV Phrap contigs were produced, which were merged by IAC into three HCMV Gap4 contigs (Table 3).

For all strains analysed, differences between the HCMV Gap4 contigs and the final sequence were few. They had several origins: errors in the HCMV Gap4 contigs resulting from IA (Table 3), poor base calling at specific locations in reads on one strand, single nucleotide polymorphisms (SNPs) and ambiguities in the lengths of tandem repeats. For tandem repeats, in some instances ambiguities were not solved uniquely for homopolymeric tracts longer than about 12 nt either by local reiterative DA or by PCR sequencing, probably because of heterogeneity in the virus genome.

Strains AD169 varUC, Towne varL, HAN13 and 3301 were processed by IAC (Table 2). Strain AD169 varUC contains a mixture of two genomes, the major one lacking a 3.7 kbp sequence consisting of the *c*′ inverted repeat and the left end of U_S_, and the minor one being intact in this region (Bradley *et al.*, 2009). The single HCMV Gap4 contig covered the major genome sequence entirely (Table 3) and contained a single error. Similarly, the HCMV Gap4 contigs obtained for strains Towne varL, HAN13 and 3301 covered the great majority of the genomes in relatively few contigs, and most contained a small number of errors.

Strains HAN20, HAN38 and 3301 were processed by IAG (Table 2). No prior sequence information was available for strains HAN20 or HAN38 and, although the strain 3301 sequence had already been determined by IAC, the IGA data were analysed afresh. The HCMV Gap4 contigs obtained for strains HAN20 and HAN38 covered the great majority of the genomes in relatively few contigs, with a small number of errors. The state of completeness of the HCMV Gap4 contigs for strain 3301 was the same as that resulting from IAC.

All of the sequences were completed by reiterative DA or limited PCR sequencing. The most prominent gaps between HCMV Gap4 contigs were located in (G+C)-rich, quasirepetitive sequences at the origin of DNA replication and in the *b*/*b*′ inverted repeat. The latter region is hypervariable, and in most cases required PCR sequencing for finishing.

### Mutations in the HCMV genomes sequenced

By comparison with sequence data available for other HCMV strains, the six strains sequenced in the present study appeared to contain the full complement of wild-type HCMV genes, with no extensive deletions. However, several apparent mutations were identified as differences from other strains that were predicted to ablate gene function (Table 2). In strain 3157, gene RL13 was frameshifted by a 2 bp insertion, and gene UL128, which consists of three coding exons, was truncated by a substitution in the first splice donor site (the crucial GT dinucleotide was mutated to CT). Both of these mutations have been reported previously from partial sequence data (Akter *et al.*, 2003). In addition, a substitution was present in the first ATG codon of the gene UL40 coding region, resulting in a change to ACG. In strain HAN13, a 17 bp deletion caused a frameshift in gene RL5A. In strain HAN20, a 35 bp deletion resulted in 3′-truncation of the gene US9 coding region. In strain HAN38, gene US9 contained an identical deletion and, in addition, a 17 bp deletion caused a frameshift in gene RL6. In strain JP, gene RL5A was frameshifted by a 2 bp deletion, and gene UL111A, which consists of three exons, was truncated by a 38 bp deletion in a region that includes the first splice-donor site.

## DISCUSSION

### Sequencing HCMV genomes

The aim of the present study was to explore the feasibility of determining HCMV genome sequences directly from infected cell cultures without purifying virus DNA or constructing bacterial plasmids and, more importantly, from clinical specimens without growing virus in cell culture. The six HCMV strains targeted included four grown in cell culture (3157 as purified virion DNA and HAN13, HAN20 and HAN38 as infected cell DNA) and two directly from clinical material (JP and 3301).

Two approaches were assessed. The first (PCR sequencing) involved amplifying HCMV genomes as sets of overlapping PCR products, sequencing them by the Sanger method, acquiring reads from a standard capillary instrument and assembling data using the Staden programs. Although it was possible to benefit from efficiencies of scale by processing strains in parallel, this approach was labour-intensive. Nonetheless, PCR sequencing yielded complete sequences for strains 3157, JP and HAN13, without prior knowledge of the target sequences or additional input from other methods. The proportions of HCMV DNA in the 3157 and JP samples are not known, and that in the HAN13 sample was estimated at 27 % (Table 3). However, substantial regions of the strain 3301 genome were easily PCR-sequenced for use in constructing a DA reference, implying that this approach would succeed for samples containing as little as 3 % HCMV DNA.

The second approach (IGA sequencing) promised greater efficiency than PCR sequencing, and involved processing IGA data by the DA or IA strategies. We had previously used DA to sequence samples of strains AD169 varUC and Towne varL, containing high proportions of DNA (92 and 47 %, respectively; Table 3). In the present study, we explored the extended capabilities of IGA sequencing using samples containing smaller proportions of HCMV DNA. DA for strains HAN13 and 3301 was successful. However, this assembly mode required a reference for each strain, and this involved prior knowledge of the target sequence. DA for strain HAN13 was straightforward, as the reference was derived from the complete genome sequence for this strain determined by PCR sequencing. However, DA for strain 3301 required greater effort because the best available reference consisted of a mosaic of 25 % strain 3301 sequences (including most of the hypervariable regions) in a backbone of 75 % strain Merlin sequences. An alternative application of DA, not requiring prior knowledge of the target genome, would involve using a generic, rather than a custom, reference. However, an attempt at DA of the strain 3301 data against a generic reference (strain Merlin) resulted in many gaps in the read consensus, due to failure to assemble reads in regions that are hypervariable or contain indels. Thus, although use of a generic reference in DA would, in principle, avoid the need to determine significant parts of the target sequence by other methods, in practice it poses a formidable hurdle. This state of affairs currently renders DA inadequate as a primary means of sequencing HCMV genomes.

IA offers obvious advantages over DA for sequencing HCMV genomes because it does not require a reference, and thus no presumptions are made about the target sequence. However, our initial experience of IA with single versions of datasets (filtered or unfiltered) was disappointing, as it resulted in large numbers of small contigs (e.g. see the outputs of individual treatments in Table 4). To overcome this, we arrived heuristically at a process for filtering reads and combining the IA contigs from multiple assemblies by Phrap assembly and then Gap4 assembly against a cognate scaffold (IAC) or a generic scaffold (IAG) to yield a high-quality reference for DA. The involvement of DA at this stage permitted the read alignment to be inspected, which, given the observation that IA contigs may contain errors, was a useful verification step. Moreover, inspection of the read alignment facilitated resolution of remaining ambiguities and gaps by local reiterative assembly or PCR sequencing.

We have shown that an HCMV genome in a sample from clinical material containing only 3 % HCMV DNA can be determined by IGA sequencing, as long as sufficient total DNA is present in the sample. The developing capability of IGA technology to produce greater numbers of longer reads in paired-end form and of the assembly software to produce more accurate, larger contigs will effectively reduce this minimum and streamline the assembly process. Moreover, future technologies are likely to improve the situation further. However, two challenges remain to the routine sequencing of complete HCMV genomes from clinical specimens. Firstly, many samples, especially those from healthy individuals, contain such low levels of HCMV DNA that they will prove refractory to sequencing without prior enrichment and amplification steps. Possibilities in this regard include the isolation of encapsidated DNA or the purification of virus DNA by buoyant density centrifugation, followed by whole-genome amplification. Secondly, many HCMV infections involve multiple strains, and methods that facilitate physical separation of their genomes, e.g. molecular hybridization via hypervariable regions, BAC cloning directly from clinical sample DNA (Zhou *et al.*, 2009) (although this suffers from the disadvantages mentioned above) or extended, single-molecule sequencing, will be required in addition.

We have now added the complete genome sequences of six HCMV strains to the three that we have already contributed to the research field. Together with the genome sequences of additional strains that we have not reported in the present paper, these will provide grist for future detailed studies of sequence variation and recombination in HCMV.

### Mutations in HCMV genomes

For the purposes of the present work, the term ‘mutation’ is used to mean a sequence difference from other strains that is predicted to ablate gene function, as opposed to variation, which, although also a result of mutational processes, is not necessarily deleterious. However, it is probable that mutations resembling variations, such as any that might be responsible for functionally detrimental amino acid replacements, would have escaped identification. Thus, the mutations identified in the strains sequenced (Table 2) were apparent because of their locations in protein-encoding regions and probable deleterious effects on functional protein expression. They included indels that cause frameshifts, and substitutions that affect initiation codons or splice sites or create termination codons. Some of the observed mutations occurred in genes RL5A, RL6 and RL13, which are hypervariable members of a group of related genes called the RL11 family.

The specific mutations in genes RL13, UL40 and UL128 that characterize strain 3157 have not been identified in other strains, and may have arisen during cell culture. Genes RL13 and UL128 (or one of its neighbours, UL130 and UL131A) often mutate during adaptation of HCMV to fibroblast cell culture (Akter *et al.*, 2003; Dolan *et al.*, 2004; Hahn *et al.*, 2004). Genes UL128/UL130/UL131A are involved in entry into non-fibroblast cells (e.g. Ryckman *et al.*, 2008), and the function of the predicted class I glycoprotein encoded by gene RL13 is not known. The presence of a substitution in the first ATG codon of the gene UL40 coding region indicates that translation occurs either from the resulting ACG codon or from the second ATG codon, or from both. This may be significant because the predicted signal peptide sequence of the glycoprotein expressed from the first ATG is similar to conserved peptides derived from cellular major histocompatibility complex class I molecules. These peptides bind to, and upregulate, expression of human leukocyte antigen E (HLA-E), which protects targets from natural killer (NK) cell lysis by interacting with CD94/NKG2A receptors. UL40 contributes to NK cell evasion by this means (Tomasec *et al.*, 2000). It would be of interest to assess the HLA-E-mediated NK evasion phenotype of strain 3157.

Neither gene RL13 nor genes UL128/UL130/UL131A are visibly mutated in the other HCMV strains grown in cell culture that were sequenced in the present study. However, each strain contains other lesions. The gene RL6 mutation in strain HAN38 has not been identified in other strains and may have arisen during cell culture. In contrast, the gene RL5A mutation in strain HAN13 is also present in the independently derived strains AD169 (Davison *et al.*, 2003) and Davis (A. J. Davison and others, unpublished data), thus indicating that it probably existed in the original clinical specimens and was inherited from a common ancestor. Also, the gene US9 mutation in strains HAN20 and HAN38 has been reported in several other HCMV isolates (Rasmussen *et al.*, 2003), and thus clearly characterizes strains that circulate in the human population. This deletion is classed as a mutation but, given its location near the 3′-end of the coding region, it is possible that it does not ablate function and therefore amounts to a variation. The role of gene RL5A during infection has not been studied, and that of gene US9 during infection is not yet known (Huber *et al.*, 2002; Mandic *et al.*, 2009).

In contrast to material from cell culture, where adaptation is known to occur, it might be expected that strains in clinical specimens would not bear mutations. Indeed, this is apparently the case for strain 3301. However, genes RL5A and UL111A are both mutated in strain JP. Gene UL111A encodes viral interleukin-10 (vIL-10; Kotenko *et al.*, 2000; Lockridge *et al.*, 2000), which has been proposed to have a role in immune modulation through its effect on myeloid cells (e.g. Chang *et al.*, 2009). Our analysis of the sequence of the BAC derived from strain PH (Table 1), which was isolated from a transplant recipient suffering from HCMV disease, identified a mutation in gene UL111A different from that in strain JP, specifically a substitution in the splice-acceptor site for the second exon (the crucial AG dinucleotide is mutated to AA). However, these findings do not necessarily indicate that HCMV strains defective in vIL-10 expression are circulating in the human population, as it is conceivable that expression of this function is not advantageous, and may even be disadvantageous, to HCMV under certain circumstances of host immunodeficiency. However, it is possible that such strains are more likely to cause disease, and thus a wider investigation of the existence of vIL-10 mutants in immunocompromised people, both with and without HCMV disease, would be worthwhile.

The observations made above have given a preliminary indication that HCMV mutants, in addition to those selected by antiviral treatment (Chou, 2008), do indeed exist in clinical settings. The finding that HCMV strains in some clinical specimens contain mutations in gene UL1 (Sekulin *et al.*, 2007), which is another hypervariable member of the RL11 gene family, is consistent with this perspective. An understanding of the extent to which HCMV mutants exist and circulate in human populations, and their potential effects on pathogenesis, is likely to be aided by further sequencing of HCMV genomes in clinical specimens.

## METHODS

### Isolation of DNA.

Pertinent features of the HCMV strains analysed are provided in Table 2. DNA for strains JP and 3301 was extracted directly from clinical specimens, and that for the other strains was obtained from cultures of human foreskin fibroblasts (HFFs) infected via clinical specimens. For strain 3157, DNA was purified from cell-released virions isolated from five 175 cm^2^ cell-culture flasks of infected HFFs as described previously (McSharry *et al.*, 2003). For strain JP, DNA was purified from 0.1 g prostate gland tissue using the NucliSENS easyMAG extraction system (bioMérieux). For strains HAN13, HAN20 and HAN38, DNA was purified from a 75 cm^2^ cell-culture flask of infected HFFs using a QIAamp 96 DNA Blood kit (Qiagen), and the numbers of virus genomes per cell were measured by standard methods at 44 417, 13 345 and 10 000, respectively (Engelmann *et al.*, 2008; Wandinger *et al.*, 2000). For strain 3301, 12 ml urine was centrifuged at 25 000 ***g*** for 1 h, and DNA was purified from the pellet as described previously (McSharry *et al.*, 2003). DNA concentrations were estimated by agarose gel electrophoresis followed by ethidium bromide staining.

### PCR sequencing.

A library of conserved, HCMV-specific primers for generating and sequencing PCR products was designed from data available for published complete HCMV genomes or substantial portions thereof (Table 1; Dolan *et al.*, 2004). The primer list is available from the corresponding author. PCR primers (usually 24 nt) were positioned so that each product overlapped its neighbours by 100–300 bp. In general, sequencing primers (usually 18 nt) were located on each DNA strand at about 500 nt intervals, so that the distance between any primer and its nearest neighbours on the opposing strand was about 250 nt. During the process of deriving genome sequences for a series of HCMV strains, primers were refined reiteratively in order to maximize conservation and effectiveness. The current number of PCR products is 105 (mean size, 2.5 kbp), and the total number of PCR and sequencing primers is 1121. Allele-specific primers were used for some hypervariable regions.

PCRs were carried out under two sets of conditions, using Advantage 2 DNA polymerase with the proprietary PCR buffer (Clontech). Details of the conditions applied to specific PCR products are available from the corresponding author. The first set of conditions was used to generate the majority of products, and involved a 50 μl reaction in 1× PCR buffer containing sample DNA (the amount being determined empirically), 0.2 μM each PCR primer (Sigma-Genosys), dNTPs (0.2 mM each) and 1 μl DNA polymerase (both from Clontech). The PCR program was conducted in a GeneAmp PCR system 9700 instrument (Applied Biosystems), and consisted of heating the reaction mixture at 95 °C for 2 min, followed by 35 cycles at 95 °C for 45 s, 60 °C for 30 s and 68 °C for 1 min kbp^−1^, and holding at 4 °C. The second set of conditions involved a 50 μl reaction in 1× PCR buffer containing sample DNA, 0.2 μM each PCR primer, dNTPs (0.2 mM each), 1.6 M betaine (Sigma-Aldrich) and 1 μl DNA polymerase. The PCR program consisted of heating the reaction mixture at 96 °C for 5 min, followed by 35 cycles at 94 °C for 15 s, 62 °C for 40 s and 68 °C for 1 min kbp^−1^, and holding at 4 °C.

The PCR products were isolated by agarose gel electrophoresis and purified by using a GENECLEAN Turbo kit (MP Biomedicals). Most PCR products were sequenced directly, but some were cloned into pGEM-T (Promega) and, for each product, four to six plasmids were sequenced.

Sequencing reactions were set up in 96-well PCR trays using proprietary reagents (Applied Biosystems). The 10 μl reactions in 0.875× PCR buffer consisted of purified PCR product (the amount being determined empirically), 0.32 μM sequencing primer (Sigma-Genosys) and 0.5 μl BigDyes (Applied Biosystems) containing DNA polymerase. The thermal program consisted of heating at 96 °C for 4 min, followed by 25 cycles at 96 °C for 10 s, 50 °C for 5 s and 60 °C for 4 min, and holding at 4 °C. Sequence data were generated at the BHF Glasgow Cardiovascular Research Centre (University of Glasgow) using an Applied Biosystems 3730 instrument.

Sequence databases were compiled from the electropherogram files by using Pregap4, Gap4 (Staden *et al.*, 2000) and Phred (Ewing & Green, 1998; Ewing *et al.*, 1998). The genome sequences were reconstructed from the final, edited databases after locating the genome termini by comparison with those determined experimentally for strain Merlin (Dolan *et al.*, 2004).

### IGA sequencing.

Sequence datasets for strains HAN13, HAN20, HAN38 and 3301, each consisting of several million single-end reads with associated quality data, were generated from DNA samples using an IGA operated according to the manufacturer's protocols at the GenePool (University of Edinburgh). Methodological information is available at http://www.illumina.com. The amount of DNA submitted for strains HAN13, HAN20 and HAN38 was approximately 1–4 μg. The strain 3301 sample contained less DNA, and a nominal amount (approx. 0.1 μg, corresponding to 2 ml urine) was submitted.

### Processing IGA data by reference-dependent assembly.

The reference consisted of an appropriate HCMV genome sequence trimmed of the inverted repeats (*ac* and *ba*) at the genome termini, except for 50 nt regions proximal to the beginning of U_L_ and the end of U_S_, in order to minimize ambiguities due to the inverted repeats. The reads were aligned with the reference using Maq-0.6.8, and the alignment of reads with the reference and read consensus was inspected by using Maqview-0.2.1 (Li *et al.*, 2008; http://maq.sourceforge.net). The reference was then improved by reiterative DA, employing the whole sequence or substrings thereof (local reiterative DA), until the final reference matched the read alignment. As a last step, the read consensus was extracted and aligned with the reference by using mafft-6.240 (Katoh *et al.*, 2005), and any differences were inspected in the read alignment. Discrepancies and gaps in the read consensus due to lack of coverage were resolved by PCR sequencing, and differences due to poor base calling at specific locations in reads on one strand or to SNPs were noted.

### Processing IGA data by reference-independent assembly.

The IGA data were processed in five stages, which are expanded below: (i) filtering the reads by a range of treatments, (ii) IA of each resulting dataset to generate contigs consisting of HCMV or cellular sequences, (iii) Phrap assembly of all of the IA contigs combined from the assemblies to generate Phrap contigs, (iv) Gap4 assembly of the Phrap contigs against an HCMV genome scaffold to generate HCMV Gap4 contigs, and (v) DA of the IGA data using a reference constructed from the HCMV Gap4 contigs. The stages of this process were enabled and connected by using Perl scripts, which are available from the corresponding author.

(i) The reads in each IGA dataset were examined for singletons (unique reads), overall quality, and quality at each base. They were filtered by removing singletons, removing reads below a minimum overall quality, truncating reads at the point where perfect quality ceased (quality truncation) or truncating reads to a set length, or combinations of these steps. The filtering treatments used for each dataset were chosen empirically on the basis of the IA output, with a preference for those that reduced the number of reads by approximately 20–60 %. Details of the filtering treatments used are listed in Table 5.

(ii) Each unfiltered or filtered dataset was assembled by using Velvet-0.7.31 (Zerbino & Birney, 2008; http://www.ebi.ac.uk/∼zerbino/velvet). Velvet uses a de Bruijn graph method to represent the reads in terms of words of hash length *k*, which is a number smaller than the read length (50 nt). The default value used was *k*=21, but *k*=25, 27 or 29 occasionally yielded longer contigs. Details of the values of *k* used are listed in Table 5. For initial assembly of each dataset, the Velvet parameters exp_cov (optimal expected coverage) and cov_cutoff (optimal coverage cut-off) were set to 1 and 0, respectively. The filtered reads were then reassembled with exp_cov optimized with the aid of functions in the R plotrix package (accessible via Velvet) and employed at all cov_cutoff values between 0 and 200. The optimal cov_cutoff value was defined as that at which the median and maximum contig sizes were greatest. The contigs (with no minimum length threshold set) were then collected for each dataset version at the optimal values of cov_cutoff and exp_cov. All IA contigs from the filtered datasets were pooled, and duplicates and substrings of other contigs were identified and removed.

(iii) The pooled, non-redundant IA contigs were assembled into larger contigs by using Phrap-1.080812 (http://www.phrap.org) with default parameters.

(iv) The Phrap contigs were aligned with an HCMV genome scaffold using Gap4, thus retaining only those contigs (HCMV Phrap contigs) that originated from the virus genome. Two alternative scaffolds were used in processes denoted IAC and IAG. For IAC, a cognate scaffold was fashioned (at least in part) from the sequence of the relevant strain determined by other methods. For IAG, a generic scaffold was constructed from the strain Merlin sequence. The HCMV Phrap contigs often exhibited clusters of sequence differences from the scaffold adjoining their ends, probably as a result of erroneous alignments that had terminated the IA contigs. These mismatched bases were removed. HCMV Phrap contigs that overlapped each other were then joined, yielding a set of HCMV Gap4 contigs.

(v) A reference consisting of the HCMV Gap4 contigs with any gaps patched by parts of the cognate or generic scaffold was used for DA. The reference was then improved and the sequence finished by reiterative DA and PCR sequencing, as described above.

## Figures and Tables

**Table 1. t1:** Published HCMV genome sequences

**HCMV strain**	**Method**	**Substrate**	**GenBank accession no.**	**Reference**
AD169 varUK	Sanger	Plasmids	BK000394	Chee *et al.* (1990)
AD169 varATCC	Sanger	BAC	AC146999	Murphy *et al.* (2003)
FIX	Sanger	BAC	AC146907	Murphy *et al.* (2003)
PH	Sanger	BAC	AC146904	Murphy *et al.* (2003)
TB40/E	Sanger	BAC	EF999921	Sinzger *et al.* (2008)
Toledo	Sanger	BAC	AC146905	Murphy *et al.* (2003)
Towne varL	Sanger	BAC	GQ121041	M. McVoy (unpublished data)
Towne varS	Sanger	BAC	AC146851	Murphy *et al.* (2003)
			AY315197	Dunn *et al.* (2003)
TR	Sanger	BAC	AC146906	Murphy *et al.* (2003)
Merlin	Sanger	Virion DNA	AY446894	Dolan *et al.* (2004)
AD169 varUC	IGA	Virion DNA	FJ527563	Bradley *et al.* (2009)
Towne varL	IGA	Infected cell DNA	FJ616285	Bradley *et al.* (2009)

**Table 2. t2:** HCMV strains analysed: sources, sequencing methods and mutations

**HCMV strain**	**Source**				Sequencing method*				**Genes mutated**
	**Clinical**	**Geographical**	**DNA**	**Passage**	**PCR**	**DA**	**IAC**	**IAG**	
3157	Urine from a congenitally infected infant	Cardiff, UK	Fibroblast culture virions	3	✓				RL13, UL40, UL128
JP	Post-mortem prostate tissue from an AIDS patient	London, UK	Clinical material	0	✓				RL5A, UL111A
AD169 varUC†	Adenoid tissue	Bethesda, MD, USA	Fibroblast culture virions	Many		✓	✓		Several
Towne varL†	Urine from a congenitally infected infant	Philadelphia, PA, USA	Fibroblast culture cells	Many		✓	✓		Several
HAN13	Bronchoalveolar lavage	Hannover, Germany	Fibroblast culture cells	3	✓	✓	✓		RL5A
HAN20	Bronchoalveolar lavage	Hannover, Germany	Fibroblast culture cells	2				✓	US9
HAN38	Bronchoalveolar lavage	Hannover, Germany	Fibroblast culture cells	2				✓	RL6, US9
3301	Urine from a congenitally infected infant	Cardiff, UK	Clinical material	0		✓	✓	✓	None

*DA, Reference-dependent assembly of IGA data; IAC, reference-independent assembly of IGA data using a cognate scaffold at the Gap4 stage; IAG, reference-independent assembly of IGA data using a generic scaffold at the Gap4 stage. ✓ indicates that the method was used. †DA of IGA data, passage histories and identities of mutated genes have been reported previously for strains AD169 varUC and Towne varL (Bradley *et al.*, 2009).

**Table 3. t3:** Results of assembling Illumina Genome Analyzer (IGA) data

**HCMV strain**	**Total reads (no.)**	DA*			IA (IAC or IAG)*						
		**HCMV reads (no.)**	**HCMV reads (%)**	**HCMV genome coverage (reads nt^−1^)**	**Total Phrap contigs (no.)**	**HCMV Phrap contigs (no.)**	**HCMV Gap4 contigs (no.)**	**HCMV genome coverage (%)**	**Gaps between HCMV Gap4 contigs (no.)**	**Total length of gaps (nt)**	**Errors in HCMV Gap4 contigs (no.)**
AD169 varUC†	6264332	5788753	92	1267	10	6	1	100	0	0	1
Towne varL†	5079235	2390159	47	516	96	9	3	99.97	2	69	0
HAN13	3157639	855653	27	184	137	19	6	99.78	5	506	7
HAN20	7035348	1040651	15	224	405	8	2	99.97	1	68	2
HAN38	7904170	591446	8	127	472	8	3	99.85	2	343	1
3301	5967563	197665	3	43	560	24	9	99.41	8	1378	1

*See Table 2 footnote. †See Table 2 footnote.

**Table 4. t4:** Results of filtering treatments for strain Towne varL

**Filtering treatment**	***k***	**Reads after filtering (no.)**	**Contig size (kb)**			**Contigs (no.)**			Velvet parameter (value)*	
			**Median**	**Maximum**	**Total**	**All**	**>1 kb**	**>5 kb**	**Exp_cov**	**Cov_cutoff**
Unfiltered	21	5079235	8	25	246	237	36	16	290	42
	25	5079235	14	44	246	178	30	13	250	41
Singletons removed	21	1682785	13	32	231	65	27	14	210	82
Overall quality:										
>1900	21	2338321	3	16	292	765	64	16	110	11
>1700	21	4156964	7	32	264	383	46	13	220	26
	25	4156964	11	32	243	150	32	16	190	44
Quality truncation, length >29	21	2609325	7	15	242	233	36	13	65	26
Truncated to length:										
40	21	5079235	14	45	251	256	21	12	190	25
	25	5079235	18	44	242	114	23	12	160	38
35	21	5079235	29	65	246	176	18	8	150	26
	25	5079235	31	68	242	99	13	10	110	30
30	21	5079235	24	68	240	90	20	11	105	32
	25	5079235	54	68	237	61	8	7	60	24

*Each dataset was assembled using Velvet-0.7.31 (see Methods). Exp_cov, Optimal expected coverage; cov_cutoff, optimal coverage cut-off.

**Table 5. t5:** Filtering treatments and hash lengths (*k*) used ✓ indicates that data from the treatment method were utilized in assembly.

**Filtering treatment**	***k***	**HCMV strain**					
		**AD169 varUC**	**Towne varL**	**HAN13**	**HAN20**	**HAN38**	**3301**
Unfiltered	21	✓	✓	✓	✓	✓	✓
	19						✓
	25	✓	✓	✓	✓	✓	✓
Singletons removed	21	✓	✓	✓	✓	✓	✓
	25	✓					
	27	✓					
Overall quality							
>1900	21		✓	✓			
>1700	21	✓	✓	✓			
	25		✓	✓			
>1300	21				✓	✓	✓
	25				✓	✓	
Quality truncation, length							
>39	21	✓		✓			✓
>29	21	✓	✓	✓			✓
Truncated to length							
49	21						✓
40	21	✓	✓	✓	✓	✓	✓
Truncated to length 40, then singletons removed	21	✓					
Truncated to length							
40	29						✓
	25		✓	✓	✓	✓	✓
35	21	✓	✓	✓	✓	✓	✓
Truncated to length 35, then singletons removed	21	✓					
Truncated to length							
35	25		✓	✓	✓	✓	
30	21	✓	✓	✓	✓	✓	✓
	25	✓	✓	✓	✓	✓	
	29	✓					

## References

[r1] Akter, P., Cunningham, C., McSharry, B. P., Dolan, A., Addison, C., Dargan, D. J., Hassan-Walker, A. F., Emery, V. C., Griffiths, P. D. & other authors (2003). Two novel spliced genes in human cytomegalovirus. J Gen Virol 84, 1117–1122. 12692276 10.1099/vir.0.18952-0

[r2] Arav-Boger, R., Zong, J. C. & Foster, C. B. (2005). Loss of linkage disequilibrium and accelerated protein divergence in duplicated cytomegalovirus chemokine genes. Virus Genes 31, 65–72. 15965610 10.1007/s11262-005-2201-3

[r3] Bates, M., Monze, M., Bima, H., Kapambwe, M., Kasolo, F. C., Gompels, U. A. & CIGNIS study group (2008). High human cytomegalovirus loads and diverse linked variable genotypes in both HIV-1 infected and exposed, but uninfected, children in Africa. Virology 382, 28–36. 18929378 10.1016/j.virol.2008.09.001

[r4] Bradley, A. J., Kovács, I. J., Gatherer, D., Dargan, D. J., Alkharsah, K. R., Chan, P. K. S., Carman, W. F., Dedicoat, M., Emery, V. C. & other authors (2008). Genotypic analysis of two hypervariable human cytomegalovirus genes. J Med Virol 80, 1615–1623. 18649324 10.1002/jmv.21241 PMC2658010

[r5] Bradley, A. J., Lurain, N. S., Ghazal, P., Trivedi, U., Cunningham, C., Baluchova, K., Gatherer, D., Wilkinson, G. W. G., Dargan, D. J. & Davison, A. J. (2009). High-throughput sequence analysis of variants of human cytomegalovirus strains Towne and AD169. J Gen Virol 90, 2375–2380. 19553388 10.1099/vir.0.013250-0 PMC2885757

[r6] Brondke, H., Schmitz, B. & Doerfler, W. (2007). Nucleotide sequence comparisons between several strains and isolates of human cytomegalovirus reveal alternate start codon usage. Arch Virol 152, 2035–2046. 17653620 10.1007/s00705-007-1026-x

[r7] Cha, T.-A., Tom, E., Kemble, G. W., Duke, G. M., Mocarski, E. S. & Spaete, R. R. (1996). Human cytomegalovirus clinical isolates carry at least 19 genes not found in laboratory strains. J Virol 70, 78–83. 8523595 10.1128/jvi.70.1.78-83.1996 PMC189790

[r8] Chang, W. L., Barry, P. A., Szubin, R., Wang, D. & Baumgarth, N. (2009). Human cytomegalovirus suppresses type I interferon secretion by plasmacytoid dendritic cells through its interleukin 10 homolog. Virology 390, 330–337. 19524994 10.1016/j.virol.2009.05.013 PMC2747589

[r9] Chee, M. S., Bankier, A. T., Beck, S., Bohni, R., Brown, C. M., Cerny, R., Horsnell, T., Hutchison, C. A., III, Kouzarides, T. & other authors (1990). Analysis of the protein-coding content of the sequence of human cytomegalovirus strain AD169. Curr Top Microbiol Immunol 154, 125–169. 2161319 10.1007/978-3-642-74980-3_6

[r10] Chou, S. (2008). Cytomegalovirus UL97 mutations in the era of ganciclovir and maribavir. Rev Med Virol 18, 233–246. 18383425 10.1002/rmv.574

[r11] Davison, A. J., Akter, P., Cunningham, C., Dolan, A., Addison, C., Dargan, D. J., Hassan-Walker, A. F., Emery, V. C., Griffiths, P. D. & Wilkinson, G. W. G. (2003). Homology between the human cytomegalovirus RL11 gene family and human adenovirus E3 genes. J Gen Virol 84, 657–663. 12604818 10.1099/vir.0.18856-0

[r12] Dolan, A., Cunningham, C., Hector, R. D., Hassan-Walker, A. F., Lee, L., Addison, C., Dargan, D. J., McGeoch, D. J., Gatherer, D. & other authors (2004). Genetic content of wild-type human cytomegalovirus. J Gen Virol 85, 1301–1312. 15105547 10.1099/vir.0.79888-0

[r13] Dunn, W., Chou, C., Li, H., Hai, R., Patterson, D., Stolc, V., Zhu, H. & Liu, F. (2003). Functional profiling of a human cytomegalovirus genome. Proc Natl Acad Sci U S A 100, 14223–14228. 14623981 10.1073/pnas.2334032100 PMC283573

[r14] Engelmann, I., Petzold, D. R., Kosinska, A., Hepkema, B. G., Schulz, T. F. & Heim, A. (2008). Rapid quantitative PCR assays for the simultaneous detection of herpes simplex virus, varicella zoster virus, cytomegalovirus, Epstein-Barr virus, and human herpesvirus 6 DNA in blood and other clinical specimens. J Med Virol 80, 467–477. 18205230 10.1002/jmv.21095

[r15] Ewing, B. & Green, P. (1998). Base-calling of automated sequencer traces using *Phred*. II. Error probabilities. Genome Res 8, 186–194. 9521922

[r16] Ewing, B., Hillier, L., Wendl, M. C. & Green, P. (1998). Base-calling of automated sequencer traces using *Phred*. I. Accuracy assessment. Genome Res 8, 175–185. 9521921 10.1101/gr.8.3.175

[r17] Hahn, G., Revello, M. G., Patrone, M., Percivalle, E., Campanini, G., Sarasini, A., Wagner, M., Gallina, A., Milanesi, G. & other authors (2004). Human cytomegalovirus UL131–128 genes are indispensable for virus growth in endothelial cells and virus transfer to leukocytes. J Virol 78, 10023–10033. 15331735 10.1128/JVI.78.18.10023-10033.2004 PMC515016

[r18] Hassan-Walker, A. F., Okwuadi, S., Lee, L., Griffiths, P. D. & Emery, V. C. (2004). Sequence variability of the *α*-chemokine UL146 from clinical strains of human cytomegalovirus. J Med Virol 74, 573–579. 15484281 10.1002/jmv.20210

[r19] Huber, M. T., Tomazin, R., Wisner, T., Boname, J. & Johnson, D. C. (2002). Human cytomegalovirus US7, US8, US9, and US10 are cytoplasmic glycoproteins, not found at cell surfaces, and US9 does not mediate cell-to-cell spread. J Virol 76, 5748–5758. 11992003 10.1128/JVI.76.11.5748-5758.2002 PMC137039

[r20] Katoh, K., Kuma, K., Toh, H. & Miyata, T. (2005). mafft version 5: improvement in accuracy of multiple sequence alignment. Nucleic Acids Res 33, 511–518. 15661851 10.1093/nar/gki198 PMC548345

[r21] Kotenko, S. V., Saccani, S., Izotova, L. S., Mirochnitchenko, O. V. & Pestka, S. (2000). Human cytomegalovirus harbors its own unique IL-10 homolog (cmvIL-10). Proc Natl Acad Sci U S A 97, 1695–1700. 10677520 10.1073/pnas.97.4.1695 PMC26498

[r22] Li, H., Ruan, J. & Durbin, R. (2008). Mapping short DNA sequencing reads and calling variants using mapping quality scores. Genome Res 18, 1851–1858. 18714091 10.1101/gr.078212.108 PMC2577856

[r23] Lockridge, K. M., Zhou, S. S., Kravitz, R. H., Johnson, J. L., Sawai, E. T., Blewett, E. L. & Barry, P. A. (2000). Primate cytomegaloviruses encode and express an IL-10-like protein. Virology 268, 272–280. 10704336 10.1006/viro.2000.0195

[r24] Lurain, N. S., Fox, A. M., Lichy, H. M., Bhorade, S. M., Ware, C. F., Huang, D. D., Kwan, S.-P., Garrity, E. R. & Chou, S. (2006). Analysis of the human cytomegalovirus genomic region from UL146 through UL147A reveals sequence hypervariability, genotypic stability, and overlapping transcripts. Virol J 3, 4 16409621 10.1186/1743-422X-3-4 PMC1360065

[r25] Mandic, L., Miller, M. S., Coulter, C., Munshaw, B. & Hertel, L. (2009). Human cytomegalovirus US9 protein contains an N-terminal signal sequence and a C-terminal mitochondrial localization domain, and does not alter cellular sensitivity to apoptosis. J Gen Virol 90, 1172–1182. 19264602 10.1099/vir.0.008466-0

[r26] Mattick, C., Dewin, D., Polley, S., Sevilla-Reyes, E., Pignatelli, S., Rawlinson, W., Wilkinson, G., Dal Monte, P. & Gompels, U. A. (2004). Linkage of human cytomegalovirus glycoprotein gO variant groups identified from worldwide clinical isolates with gN genotypes, implications for disease associations and evidence for N-terminal sites of positive selection. Virology 318, 582–597. 14972526 10.1016/j.virol.2003.09.036

[r27] McSharry, B. P., Tomasec, P., Neale, M. L. & Wilkinson, G. W. G. (2003). The most abundantly transcribed human cytomegalovirus gene (*β*2.7) is non-essential for growth *in vitro*. J Gen Virol 84, 2511–2516. 12917473 10.1099/vir.0.19298-0

[r28] Murphy, E., Yu, D., Grimwood, J., Schmutz, J., Dickson, M., Jarvis, M. A., Hahn, G., Nelson, J. A., Myers, R. M. & Shenk, T. E. (2003). Coding potential of laboratory and clinical strains of human cytomegalovirus. Proc Natl Acad Sci U S A 100, 14976–14981. 14657367 10.1073/pnas.2136652100 PMC299866

[r29] Pignatelli, S., Dal Monte, P., Rossini, G. & Landini, M. P. (2004). Genetic polymorphisms among human cytomegalovirus (HCMV) wild-type strains. Rev Med Virol 14, 383–410. 15386592 10.1002/rmv.438

[r30] Prichard, M. N., Penfold, M. E. T., Duke, G. M., Spaete, R. R. & Kemble, G. W. (2001). A review of genetic differences between limited and extensively passaged human cytomegalovirus strains. Rev Med Virol 11, 191–200. 11376481 10.1002/rmv.315

[r31] Puchhammer-Stöckl, E. & Görzer, I. (2006). Cytomegalovirus and Epstein-Barr virus subtypes – the search for clinical significance. J Clin Virol 36, 239–248. 16697698 10.1016/j.jcv.2006.03.004

[r32] Rasmussen, L., Geissler, A. & Winters, M. (2003). Inter- and intragenic variations complicate the molecular epidemiology of human cytomegalovirus. J Infect Dis 187, 809–819. 12599055 10.1086/367900

[r33] Ryckman, B. J., Chase, M. C. & Johnson, D. C. (2008). HCMV gH/gL/UL128–131 interferes with virus entry into epithelial cells: evidence for cell type-specific receptors. Proc Natl Acad Sci U S A 105, 14118–14123. 18768787 10.1073/pnas.0804365105 PMC2544588

[r34] Sekulin, K., Görzer, I., Heiss-Czedik, D. & Puchhammer-Stöckl, E. (2007). Analysis of the variability of CMV strains in the RL11D domain of the RL11 multigene family. Virus Genes 35, 577–583. 17823862 10.1007/s11262-007-0158-0

[r35] Sinzger, C., Hahn, G., Digel, M., Katona, R., Sampaio, K. L., Messerle, M., Hengel, H., Koszinowski, U., Brune, W. & Adler, B. (2008). Cloning and sequencing of a highly productive, endotheliotropic virus strain derived from human cytomegalovirus TB40/E. J Gen Virol 89, 359–368. 18198366 10.1099/vir.0.83286-0

[r36] Staden, R., Beal, K. F. & Bonfield, J. K. (2000). The Staden package, 1998. Methods Mol Biol 132, 115–130. 10547834 10.1385/1-59259-192-2:115

[r37] Stanton, R., Westmoreland, D., Fox, J. D., Davison, A. J. & Wilkinson, G. W. G. (2005). Stability of human cytomegalovirus genotypes in persistently infected renal transplant recipients. J Med Virol 75, 42–46. 15543586 10.1002/jmv.20235

[r38] Tomasec, P., Braud, V. M., Rickards, C., Powell, M. B., McSharry, B. P., Gadola, S., Cerundolo, V., Borysiewicz, L. K., McMichael, A. J. & Wilkinson, G. W. G. (2000). Surface expression of HLA-E, an inhibitor of natural killer cells, enhanced by human cytomegalovirus gpUL40. Science 287, 1031–1033. 10669413 10.1126/science.287.5455.1031

[r39] Wandinger, K.-P., Jabs, W., Siekhaus, A., Bubel, S., Trillenberg, P., Wagner, H.-J., Wessel, K., Kirchner, H. & Hennig, H. (2000). Association between clinical disease activity and Epstein-Barr virus reactivation in MS. Neurology 55, 178–184. 10908887 10.1212/wnl.55.2.178

[r40] Yan, H., Koyano, S., Inami, Y., Yamamoto, Y., Suzutani, T., Mizuguchi, M., Ushijima, H., Kurane, I. & Inoue, N. (2008). Genetic linkage among human cytomegalovirus glycoprotein N (gN) and gO genes, with evidence for recombination from congenitally and post-natally infected Japanese infants. J Gen Virol 89, 2275–2279. 18753237 10.1099/vir.0.83685-0

[r41] Zerbino, D. R. & Birney, E. (2008). Velvet: algorithms for *de novo* short read assembly using de Bruijn graphs. Genome Res 18, 821–829. 18349386 10.1101/gr.074492.107 PMC2336801

[r42] Zhou, F., Li, Q. & Gao, S. J. (2009). A sequence-independent in vitro transposon-based strategy for efficient cloning of genomes of large DNA viruses as bacterial artificial chromosomes. Nucleic Acids Res 37, e2 18988631 10.1093/nar/gkn890 PMC2615602

